# Key crops for processed foods have spatially variable biodiversity impacts not captured in other environmental impact indicators

**DOI:** 10.1038/s41598-025-34850-2

**Published:** 2026-01-29

**Authors:** Felicitas Pamatat, Charlotte L. Outhwaite, Amy Molotoks, Carole Dalin, Mark Jwaideh, Abbie S. A. Chapman

**Affiliations:** 1 School of Biological Science, University of Aberdeen, Aberdeen, UK; 2 Centre for Biodiversity & Environment Research, University College London, Gower Street, London, WC1E 6BT UK; 3 Institute of Zoology, Zoological Society of London, Outer Circle, Regent’s Park, London, NW1 4RY UK; 4 Stockholm Environment Institute, University of York, York, UK; 5 UCL Institute for Sustainable Resources, University College London, 14 Upper Woburn Place, London, WC1H 0NN UK; 6 Laboratoire de Géologie, École Normale Supérieure, CNRS, PSL Université, IPSL, Paris, France; 7 Environmental Change Institute, University of Oxford, Oxford, UK

**Keywords:** Sustainable, Food system, Biodiversity, Environmental impact assessment, Consumption, Trade, Ecology, Ecology, Environmental sciences

## Abstract

We must urgently address unsustainable food system practices to protect important ecosystems. Most research quantifying the environmental impacts of food production and consumption has not measured biodiversity impacts directly or spatially, limiting our ability to evaluate how producing and consuming countries are contributing to food system sustainability. Here, we quantify the biodiversity impacts of food crops commonly found in processed foods using a range of accessible metrics. We focus on ingredients found in popular processed foods, such as chocolate digestive biscuits, and show globally reaching impacts. Land-use and fertiliser impacts were greatest for the food crops considered. We also identified Critically Endangered species at risk from habitat loss associated with crops being supplied to the UK specifically. For instance, cocoa production overlaps with nearly 20% of the range of both the Critically Endangered Roloway and Miss Waldron’s Red Colobus monkeys. As crop-specific farm and trade data are not publicly available, it is difficult to know the origin of all ingredients, limiting biodiversity impact estimation. If such data are released in future, our approach can be used to further evaluate biodiversity impacts of foods to inform more sustainable decision-making at consumer, business, and government levels.

**Supplementary Information:**

The online version contains supplementary material available at 10.1038/s41598-025-34850-2.

## Introduction

Food production is a major driver of biodiversity decline^1–3^ due to the shift of land use from natural lands towards agriculture, the unsustainable intensification of commodity production, and the subsequent loss of habitats and species^4–6^. Of the Earth’s habitable land surface, approximately 10% is used for cropland and 37% for livestock feed and grazing^7^. With the global human population projected to reach 9.7 billion by 2050^7,8^, there is a trade-off between land required for food production and for biodiversity conservation^9,10^. This necessitates increasing the sustainability of food production worldwide, to ensure that both agricultural and conservation-based benefits can be provided into the future^11,12^.

Dependence on imports and international supply chains which involve numerous actors, as well as differing agricultural practices between crop groups^13^, means that creating sustainable food-production pathways is complex. As such, there is considerable variability in the impacts associated with the production of each food item^13,14^. Several recent studies have assessed the environmental impacts of specific food products, including assessments of water use, carbon production, and land use^14–19^. For example, Poore and Nemecek^14^ used a meta-analytical approach to highlight the variability in environmental impacts associated with different foods (e.g., rice, potatoes, nuts, citrus). Similarly, Clark et al.^16^ used open data to estimate the impacts of 57,000 food products on greenhouse gas emissions, land use, water stress, and eutrophication potential, which found that more nutritious foods also tend to be more environmentally sustainable. Recently, Jwaideh and Dalin^15^ developed a single, multidimensional index of the environmental impacts of crop production, considering water stress and biodiversity loss due to greenhouse gas emissions, water consumption, land occupation, and fertiliser application.

Most research focusing on specific foods or diets does not include effects on biodiversity as a measure of sustainability or environmental impact. It is also uncommon for impacts to be represented spatially, showing where crops are produced and where the demand for those crops originates, limiting our understanding of the contributions of different countries to sustainable development (though notable exceptions include Jwaideh and Dalin^15^, Halpern et al.^13^, and Chaudhary and Kastner^2^). Furthermore, trade-offs and synergies exist among measures of environmental sustainability. For instance, where carbon footprints are low, biodiversity impacts could be high, making it challenging to assess the environmental sustainability of consumption, especially if studies focus on specific impact metrics or group multiple measures into a single index.

Here, we quantify the environmental impacts associated with the key ingredients of processed foods, using biscuits as an illustrative example. Focusing on specific ingredients enables us to explore the challenges of understanding sustainable food systems, including representing biodiversity impacts alongside other impact-based metrics, the variation in impact across space, and novel ways to communicate these impacts to consumers. We focus part of our study on the United Kingdom (UK), as biscuits have become both a cultural norm and a staple (e.g., included as an ‘essential’ in Food Bank lists and among Trussell Trust’s most requested items^20^) and named an ‘affordable treat’^21^). An estimated twelve billion biscuits are consumed in the UK per year (estimate based on the weight of a McVities dark chocolate digestive stated publicly as 16.7 g and the weekly UK household consumption of biscuits from 2006 to 2020 on statista.com and UK 2019 population estimate from ons.org.uk). Biscuits are generally made using three main ingredients: wheat, sugar, and palm oil, which are also common in many other low-cost, processed foods and foods considered ‘treat’ foods. Biscuits vary in composition and can include or exclude chocolate, so there is scope for more sustainable consumer decision-making even within this product group if chocolate (cocoa) has strong environmental impacts. As well as providing a useful entry point for raising consumer awareness of differences in product sustainability and the challenges associated with quantifying this, biscuits and processed goods in general are commonly excluded from biodiversity-impact studies, where the focus instead tends to be on general food groups such as meat, dairy, fruits and vegetables, and nuts (though see Konstantas et al., 2019^22^, 2018^23^). Our work addresses this gap. Using crop distribution, trade, and environmental impact data, we: (i) summarise and map the global scale environmental impacts of each of the four crops (wheat, sugar, palm oil and cocoa), identifying hotspots of high impact (Q1); (ii) compare the impacts in the countries from which the UK sources the majority of these crops (Q2); and (iii) investigate the extent to which threatened mammal species could be impacted by the production of these crops (Q3). As well as highlighting the international impacts of processed goods consumed in the UK, our findings can be used as a communication tool to bridge the gap between knowledge of potential impacts and consumer understanding of those impacts to support better-informed sustainable decision-making.

## Methods

### Global-scale environmental impacts of wheat, sugar, palm oil, and cocoa

**Fig. 1:**
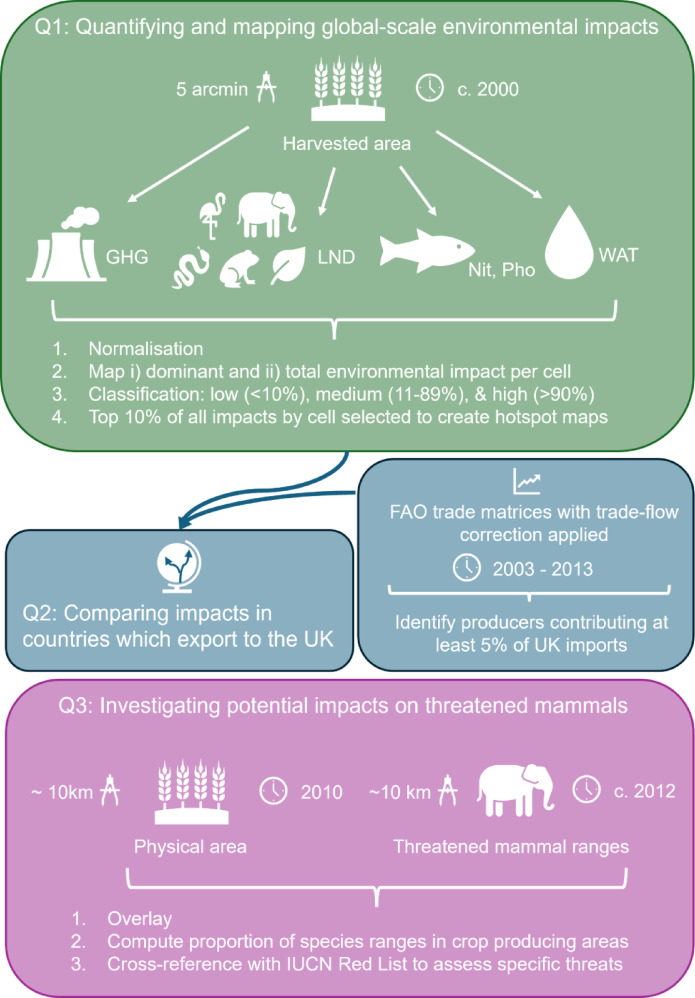
Using global-scale maps representing five environmental impact indicators for four key crops (wheat, sugar, cocoa and oil palm), we assessed three questions (Q1-Q3) relating to sustainable crop production. GHG refers to greenhouse gas emissions in tonnes of carbon dioxide equivalent per year. LND refers to the occupational land use biodiversity impacts, Nit—nitrogen fertiliser impacts on marine demersal fish and invertebrate species, and Pho—phosphorus impacts on freshwater fish species, all expressed as Potentially Disappeared Fraction of species per year, per tonne of crop. WAT is the water debt, measuring the sustainability of water use in years.

In brief, we first summarised the global-scale environmental impacts of four focal crops (wheat, sugar, oil palm, and cocoa) commonly used in biscuits and other processed foods using global-scale indicators of environmental impact, based on: greenhouse gas emissions (GHGs; Carlson et al.^24^), land use (Jwaideh and Dalin^15^), water debt (Tuninetti et al.^25^), and fertiliser application (nitrogen impacts on marine biodiversity and phosphorus impacts on freshwater fish; Jwaideh et al.^26^). Using the UK as a case study, we then assessed the environmental impacts of these four crops in the regions from which they are sourced to supply the UK, identified using international food trade data. Finally, to complement the measures of environmental impact already quantified, we extended our analysis to consider especially high-risk regions by overlaying range maps of threatened mammal species^27^ with maps of crop production in countries from which the UK sources the most substantial quantities of each crop (Fig. 1).

### Global scale maps of the environmental impact of crop production

We used five environmental impact indicators which were developed using spatially explicit environmental models related to climate change, water stress, and terrestrial and aquatic biodiversity loss. Below, we give a brief outline of each metric and of further processing applied. We recommend referring to Carlson et al.^24^; Tuninetti et al.^25^; Chaudhary et al.^28^; and Jwaideh et al.^26^, respectively, for a more detailed explanation of their development. We mapped the impacts of all metrics resulting from the four key crops production using ‘ggplot2’^29^ and ‘raster’^30^ in R^31^. We make use of available maps for each of the five indicators at a 5 arc-minute resolution and global extent for each of our four crops of interest (though only two indicators could be mapped for cocoa using available data). Each indicator is centered on data from around the year 2000 as this most closely matched the high resolution, spatially explicit, global-scale and crop-specific data (harvested area, yield, fertiliser application, irrigated area) available at the time^32,33^. Dry-weight production values per grid cell, in tonnes, were calculated using dry-matter fractions in Bouwman et al.^34^ which were multiplied by production data from Monfreda et al.^32^.

### Harvested-area data

Data on the global harvested area of each of the crops (wheat, cocoa, sugarcane and sugar beet, and oil palm) were sourced from Monfreda et al.^32^. These data, which combine cropland data with national and subnational census statistics, are at a 5-arc minute resolution (approximately 9 km by 9 km at the equator) and provide the harvested area, production (tonnes), and yield values for 172 crops and groups of crops (such as berries, citrus, etc.^32^). The data represent around the year 2000, but were selected for indices of environmental impact because of the comprehensive suite of crops represented at subnational level (e.g., relative to 42 crops available in MapSPAM^35^) and to ensure temporal coincidence with the fertiliser data, which were only available for phosphorus for the year 2000. These data, which combine census data from multiple spatial scales (e.g., national, state, county) with cropland data using a data fusion modelling approach, cannot be used to comment on values in specific grid cells^32^.

To complement environmental impact analyses using these data, we conducted analyses specific to threatened species using more recent global harvested-area data for 2010 for cocoa, sugar, wheat, and oil palm from MapSPAM, the International Food Policy Research Institute (IFPRI) Spatial Production Allocation Model^35,36^. Note that here we use the term ‘oil palm’ to refer to the crop plant and ‘palm oil’ to refer to the ingredient or product from this plant. In datasets, this will often be ‘oilpalm’. The threatened species range data represent circa 2012, so 2010 crop data were better temporally aligned for this part of our analysis.

### Greenhouse gas emissions (GHG)

Total greenhouse gas emission intensity metrics (GHG) were quantified in tonnes of carbon dioxide equivalent per year based on Carlson et al.^24^. This metric estimates impact based on the GHG emissions (methane, carbon dioxide, and nitrous oxide) of rice paddy management, peatland draining, and fertiliser application, but not land-use change. We multiplied the GHG by dry-weight production per grid cell, in tonnes, to quantify the GHG impact per grid cell, without the impact of the differing water content of different crops. The GHG measure is not a direct measure of biodiversity impact, but will impact biodiversity via climate change, habitat loss, and increasing risk of species extinctions. The key assumptions of this dataset, including peat coverage assumed in areas marked as peatlands, and manure applied within the grid cell within which it was produced, are described in detail in Carlson et al^24^.

### Occupational land use biodiversity impacts (LND)

The impacts of land use/degradation on mammal, bird, reptile, amphibian, and vascular plant species were estimated using the occupational land use biodiversity impacts measure (LND) described in Chaudhary et al.^28^. We used these data as they form the basis of a multi-dimensional environmental impact indicator proposed for use by industry for corporate sustainability measures^15^, and could reasonably be assumed to be used as a metric of biodiversity impact by the food industry. These data use information on the relative change in species richness in different land uses relative to a natural regional reference, which is then used to model species extinctions due to land-use change. The unit of this measure is Potentially Disappeared Fraction of species per year, per tonne of crop. We produced impact values per grid cell by multiplying these values by dry-weight production per grid cell, in tonnes. We note here the limitation that amphibians and reptiles had less complete input data than available for plants, mammals, and birds, as is still the case generally, and some biomes are less well represented than others, so these data should be used only for interpretation on large scales, such as presented herein, rather than for specific, within-country biodiversity impacts. Chaudhary et al. acknowledge these limitations and note that their products could underestimate the impacts on biodiversity of land use^28^.

### Impacts of nitrogen on marine species (Nit) and of phosphorus fertilisers on biodiversity (Pho)

Indicators of the impacts of nitrogen fertiliser (Nit, abbreviated as such to distinguish the impact indicator from the N for nitrogen more generally) on marine demersal fish and invertebrate species and of phosphorus fertiliser on freshwater fish species (Pho, abbreviated as such to distinguish the impact indicator from the P for phosphorus more generally) were calculated as Potentially Disappeared Fraction of species per year, per tonne of crop, using the method detailed in Jwaideh et al.^26^. This approach incorporates a spatially explicit nutrient fate and transport model, with a receptor exposure and effect model to determine the biodiversity impacts of 17 crops on each system linked to the cropland soil emissions via the watershed^26^. The data do not consider fertiliser emissions and aquatic biodiversity impacts associated with crop production along the supply chain, only at the farm stage, and cannot account for differences in farming techniques at this time. Values per grid cell were calculated by multiplying the Nit and Pho data by dry-weight production in tonnes.

### Water debt (WAT)

To measure the impact of the production of each crop on water sustainability, we used the ‘water debt’ measure (unit: years) detailed in Tuninetti et al.^25^. This is not a direct measure of biodiversity impact but will impact biodiversity via water available to wild plants, freshwater species, and other fauna. The water debt indicator is available at 5 arc minute spatial resolution for the year 2000 (coherent with the crop distribution data from Monfreda et al.^32^, used in our analysis). Water debt is calculated using the ratio of the crop-, water-source-, and location-specific annual crop water footprint in a given cell to the average renewable amount of water available in the cell each year, where the renewability rate is based on a long-term average renewability rate from 1987 to 2013. When the water debt value is greater than one year, the annual crop production is unsustainable relative to the local availability of water. In this metric, environmental flow requirements are not removed from the availability of surface water, which overestimates water availability for irrigation^25^. Conversely, the availability of water is only based on the runoff generated from rainfall in the grid cell, so it excludes upstream flow, underestimating actual surface water availability for irrigation^25^. These two assumptions could compensate each other in some regions^25^. For more detailed information on this measure and its methodology, see Tuninetti et al.^25^.

For our analysis, we only considered WD values greater than one (indicating unsustainability), to ensure we considered areas where water use is unsustainable and is therefore likely to put pressure on biodiversity. By taking only measurements that could be considered unsustainable (values above one), this metric aligns with the other metrics that represent an impact on biodiversity or the environment at positive values, on a continuous scale. It is possible that some water stress will occur at values below 1, which may result in an underestimation of water debt impacts in some areas. As the values for water debt for all sources can only exceed one where there is groundwater use (overuse) or if the surface water use relies on precipitation falling in upstream grid cells, this means that we are representing areas which have been irrigated and do not consider appropriation of water from soil moisture in this measure.

### Mapping areas of greatest environmental impact

We used maps of environmental impact per grid cell for greenhouse gas emissions (GHG), occupational land use biodiversity impacts (LND), phosphorus (Pho), nitrogen (Nit), and water debt (WAT), to identify areas of greatest impact for each of our study’s four focal crops (wheat, sugar, oil palm, and cocoa). For cocoa, only greenhouse gas emissions (GHG) and occupational land use biodiversity impact (LND) values were available. Sugar data were created by overlaying the sugarcane and sugar beet datasets for both the impact maps and the harvested-area map. Although mostly cultivated in different regions, to account for any overlap of the sugarcane and sugar beet dataset, the values of impact or harvest in occupied cells were summed. Zeros in all impact factor maps were replaced with NA, as the zeros were predominantly in areas of water bodies and were therefore present because terrestrial impacts were being measured rather than representing genuine zero values. We used scaling normalisation (minimum–maximum) to ensure all impact factor values were comparable across indicators. Notably, so we did not lose information on areas of unsustainable water debt while normalising the data, any values below one were removed before normalising, as values below one are deemed sustainable (less water is used than annually available from the source in the cell^25^, although it could be missing unsustainable cases where environmental flow requirements are not satisfied) and we focused on regions with water debt (unsustainable use, with values greater than one).

We categorised environmental impact values into low (< 10%), medium (11–89%), and high-impact (> 90%) classes and identified areas with the highest impact values as the top 10% of all impacts by cell. By filtering and selecting the highest impact areas, we were able to pinpoint regions with significant environmental concerns. To provide insights into the spatial distribution of each impact, and trade-offs between them, we created individual hotspot maps for GHG, LND, Pho, Nit, and WAT using the high-impact class. In addition to the individual impact maps, two combined maps were created: (i) identifying the dominant environmental factor for each cell; and (ii) mapping the total environmental impact, calculated by summing all values of the environmental indicators for each cell. We took this approach to determine the regions with the greatest environmental impacts across all available indicators (Q1).

As a simpler, consumer-friendly and complementary presentation of potential regions of trade-off, we also mapped areas of overlap between high species richness and harvested area. We use species richness as a measure of biodiversity because richness data are most readily available at the global scale for the greatest number of vertebrates (here, focusing on terrestrial mammals, birds, reptiles, and amphibians). We focus on vertebrates, as these are the best-sampled taxonomic groups on a global scale. We used spatialised species-richness estimates from Etard et al.^37^, computed at 50-km resolution (27 arc minutes) on the global scale using species range-distribution maps from BirdLife International^38^ and the IUCN^39^, excluding areas outside of known elevational limits for each species. The species-richness maps, which included separate maps for amphibians, mammals, birds and reptiles, were given a Behrmann projection. To ensure that all data layers were aligned, the harvested area maps were resampled to match the species-richness maps’ lower resolution and cropped according to the maximum and minimum spatial extent.

We used the ‘ggplot2’^29^, ‘dplyr’^40^, ‘tidyr’^41^, ‘classInt’^42^, ‘magrittr’^43^, ‘raster’^30^, and ‘patchwork’^44^ R packages and R 4.0.0^31^ for the visualisation and data preparation.

### Comparing the environmental impacts of countries exporting cocoa, sugar, wheat, and palm oil to the UK

We assessed the total impact across all environmental indicators for the focal crops in the countries from which the UK imports them (Q2). To do this, we considered the source countries and the quantities of wheat (including wheat products), sugar (sugar beet and sugarcane), palm oil, and cocoa (including beans, butter, paste, powder and cake) imported to the UK between 2003 and 2013. FAOSTAT trade matrices (FAOSTAT; http://www.fao.org/faostat/en/#data/TM) were used to determine the trade of each crop and products from each partner country to the UK. These years were selected to best match the crop data (c.2000) and species-richness data (c.2012) available. These data are also representative of recent trade, as opposed to more recent years that are affected by the Covid-19 pandemic and reporting lags. A trade-flow-correction was used to re-assign trade flows to their origin country when they are re-exported, using an origin-tracing algorithm developed in Kastner et al.^45^ and updated and applied in Dalin et al.^46^. Using these data, we identified the producers contributing at least 5% of the UK’s imports of the four focal crops (including UK-UK trade, here termed ‘domestic production’). We combined sugarcane and sugar beet in trade data before consequent analyses, as many items in the source data (e.g., refined sugar) do not specify the crop, so this was the most conservative approach.

Using this list of countries (Table 1) and the maps of crop-specific environmental impact for each of the five indicators, we assessed and compared the environmental impacts of the crops within each of the source countries.

**Table 1:** Countries that supplied at least 5% of the UK’s consumption of wheat, cocoa, sugar and palm oil. Total supplied is the tonne weight of the total crop imported into the UK, on average, between 2003 and 2013, and the percentage of UK consumption this represents. Note that sugar was assessed by including both sugarcane and sugar beet sources; however, the majority for the UK is domestic sugar beet production. Also presented is the percentage of the exporting country’s total production for that crop. Data sourced from FAO (FAOSTAT; http://www.fao.org/faostat/en/#data/TM) and adjusted to re-assign trade flows to origin countries rather than representing re-exports using an algorithm created by Kastner et al.^45^. A number of other countries supply less than 5% of the UK’s total imports of these crops but we chose to focus on those that supply the majority. The full list of source countries is provided in Supporting Information.

Crop	Country	Total supplied (tonnes)	Percentage of UK consumption (%)	Percentage of country’s production exported to the UK
Wheat	United Kingdom	14,030,964	87	NA
Cocoa	Côte d’Ivoire	65,916	36	4.7%
	Ghana	64,620	35	9%
	Nigeria	19,292	10	4.8%
	Cameroon	12,280	7	5.1%
Sugar	United Kingdom (sugar beet)	7,276,708	92	NA
Oil palm	Indonesia	1,944,456	46	1.8%
	Malaysia	1,787,703	43	2.2%

### Evaluating the potential pressures being placed on threatened species by cocoa, sugar, wheat, and palm oil

Focusing on terrestrial mammals, we quantified the potential for pressure on threatened species according to the IUCN Red List^27^ associated with the four focal crops (Q3). We focused on mammals as they are a well-studied and well-known group, so assist in our goal of identifying effective ways to communicate sustainability and impact. We also concentrated on the countries providing most of the cocoa, palm oil, sugar and wheat supply to the UK (Table 1).

We used the International Food Policy Research Institute’s (IFPRI) Spatial Production Allocation Model (also known as MapSPAM)^35^, which generates global estimates of crop distributions and provides high-resolution maps (5 arc minute) of 42 crops. We used the physical area where crops are grown in 2010 for this analysis. Although more recent data are now available, we used a version of MapSPAM which is more closely aligned with the time covered by species-range data (c. 2012). By summing species ranges (from the IUCN^47^) for mammals within each pixel, we assume that a species is present across its entire range and compute a spatialised estimate of species richness in 10-km grid cells across the globe.

To measure the relative potential pressure placed on threatened mammal species by cocoa and oil palm, we overlaid crop area maps with maps of threatened species ranges using Google Earth Engine^48^. We then computed the proportion of threatened species ranges found within the producing areas of each focal crop to quantify the relative pressure placed on species threatened by habitat loss (potentially due to agricultural expansion). Spatialised results were cross-referenced with information on specific threats to individual Red List species. This analysis was performed for the categories: Critically Endangered (CR), Endangered (EN), and Vulnerable (VU). We show results in this paper only for the highest threat category (CR), but all are provided in the Supporting Information.

We took a different approach for wheat and sugar because most of these crops are supplied domestically within the UK. There are no threatened mammal species, according to the IUCN Red List, in the UK. Therefore, we used *Sciurus vulgaris* (the red squirrel) as an example for our case study. Although this species is given the ‘Least Concern’ status by the IUCN Red List globally, red squirrels are considered threatened in the UK. They are a priority species under the UK Biodiversity Action Plan and are the only species listed as endangered by The Mammal Society’s Red List for Britain’s Mammals^49^. Spatial distribution data are available for this species from the IUCN^47^, so we overlaid the red squirrel and crop-area data to produce a map showing harvest values within the red squirrels’ range.

## Results

**Fig. 2:**
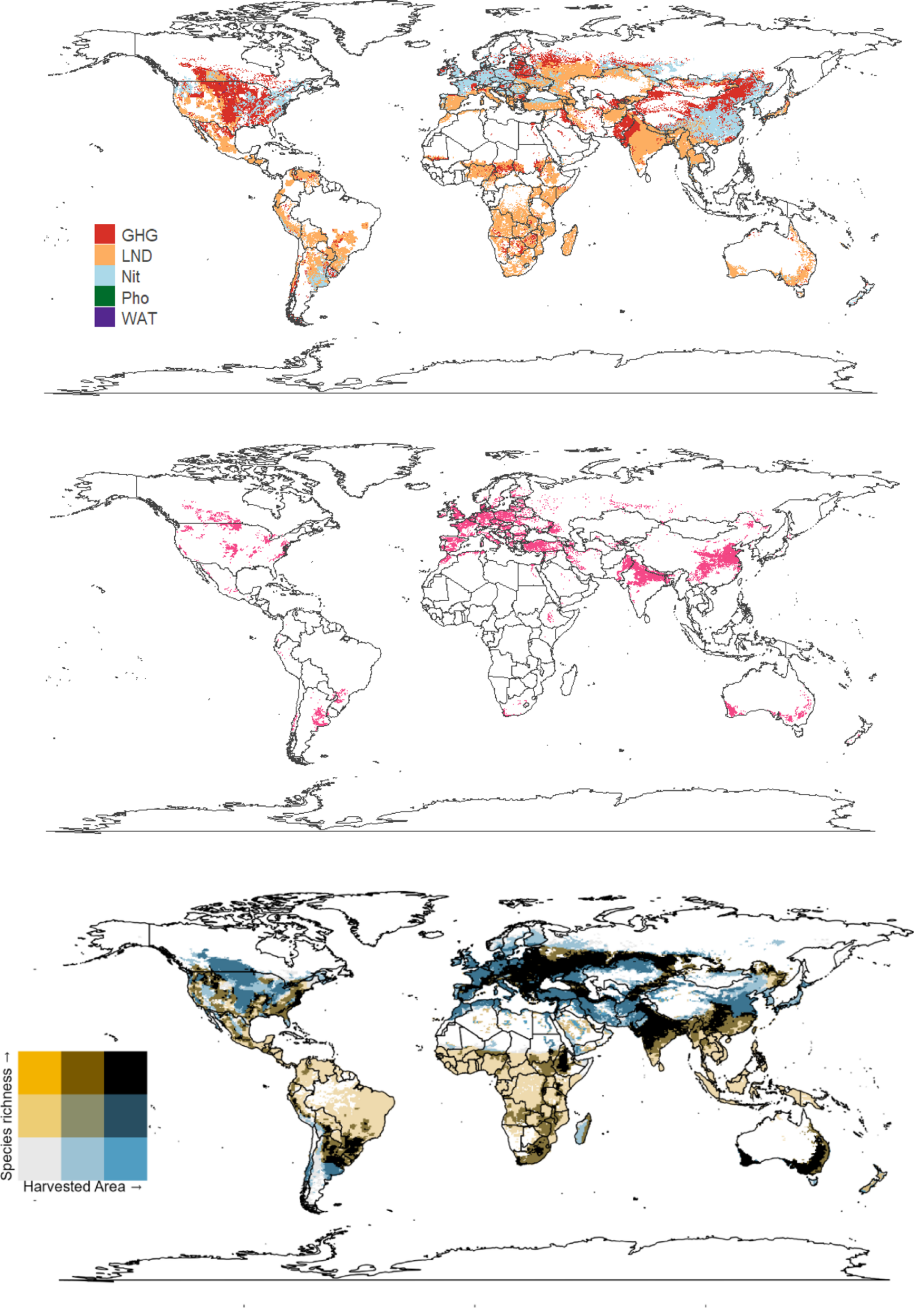
The impact of wheat production on: greenhouse gas emissions (GHG), occupational land use biodiversity (LND), marine demersal fish and invertebrate species via nitrogen fertiliser application (Nit), freshwater fish species via phosphorus (Pho) fertiliser application, and the water debt (WAT); (**a**) highest impact within each cell; (**b**) top 10% of areas with the highest summed impact values; (**c**) bivariate map of harvested area and species richness. These maps were generated using the ‘raster’^30^ and ‘ggplot2’^29^ R packages with a ‘maps’ package^50^ world outline.

**Fig. 3:**
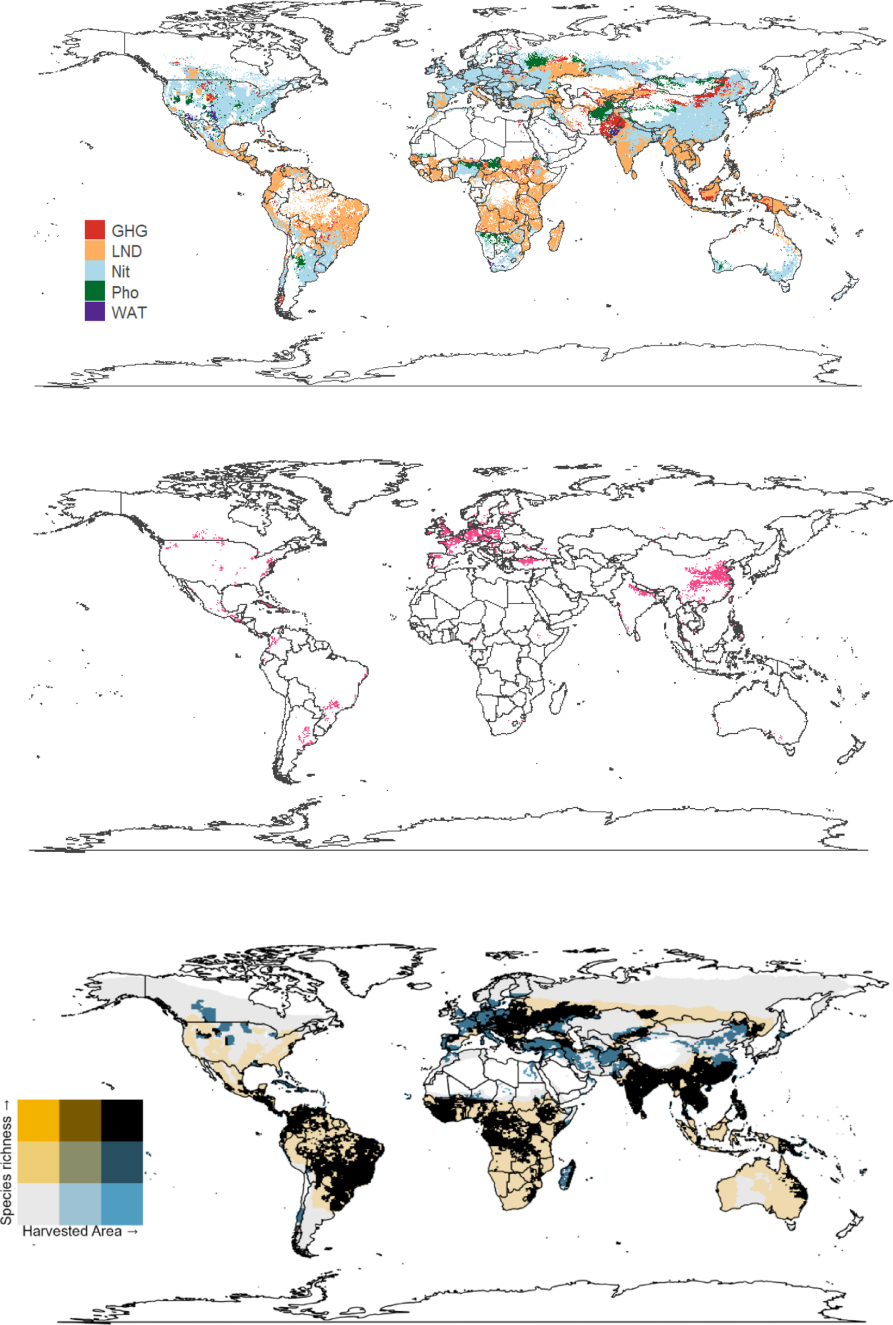
The impact of sugar (sugarcane and -beet combined) on: greenhouse gas emissions (GHG), occupational land use biodiversity (LND), marine demersal fish and invertebrate species via nitrogen fertiliser (Nit), freshwater fish species via phosphorus (Pho), and the water debt (WAT); (**a**) highest impact within each cell; (**b**) top 10% of areas with the highest summed impact values; (**c**) bivariate map of harvested area and species richness. These maps were generated using the ‘raster’^30^ and ‘ggplot2’^29^ R packages with a ‘maps’ package^50^ world outline.

**Fig. 4:**
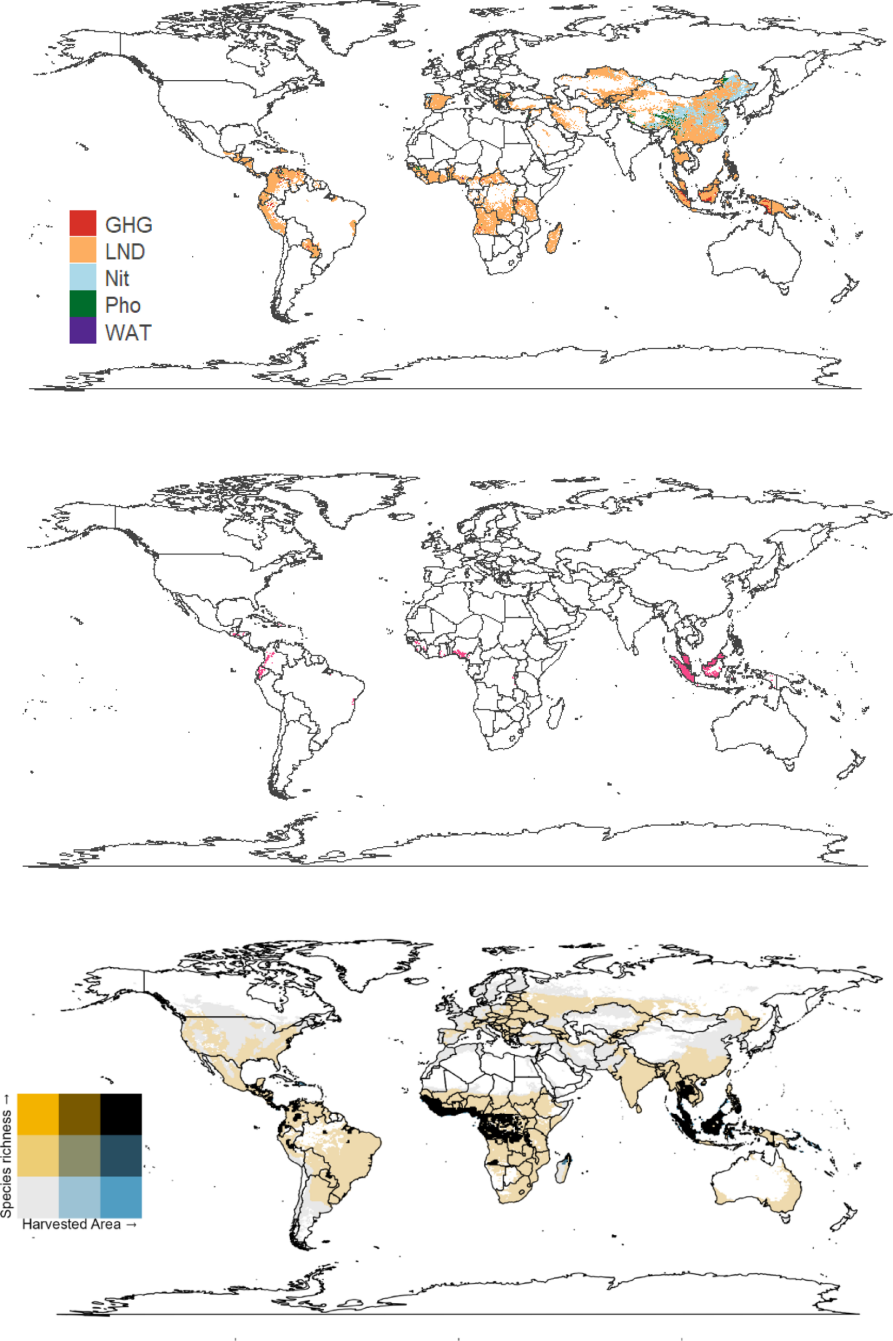
The impact of oil palm on: greenhouse gas emissions (GHG), occupational land use biodiversity (LND), marine demersal fish and invertebrate species via nitrogen fertiliser (Nit), freshwater fish species via phosphorus (Pho), and the water debt (WAT); (**a**) highest impact within each cell; (**b**) top 10% of areas with the highest summed impact values; (**c**) bivariate map of harvested area and species richness. These maps were generated using the ‘raster’^30^ and ‘ggplot2’^29^ R packages with a ‘maps’ package^50^ world outline.

**Fig. 5:**
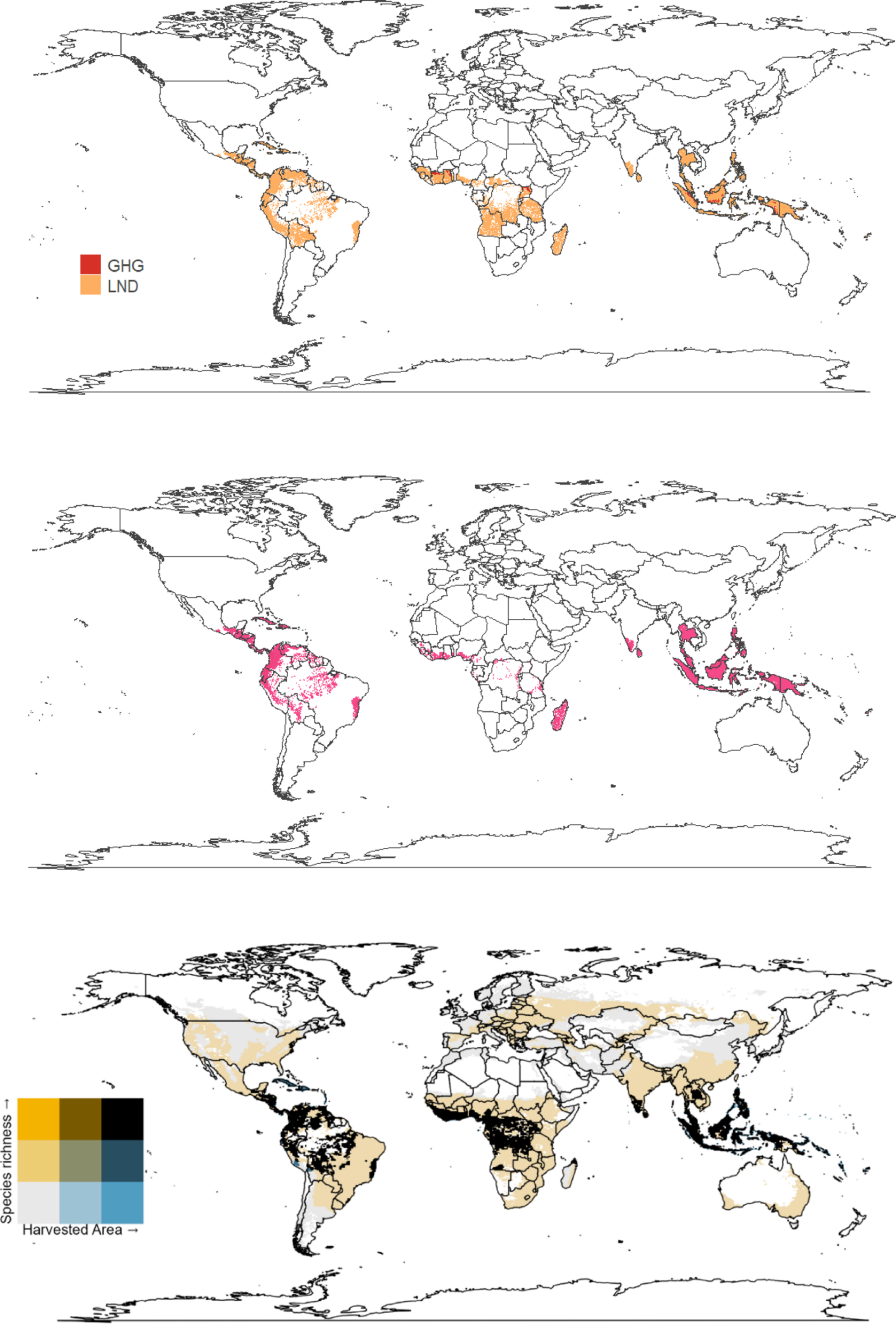
The impact of cocoa on greenhouse gas emissions (GHG) and occupational land use biodiversity (LND); (**a**) highest impact within each cell; (**b**) top 10% of areas with the highest summed impact values; (**c**) bivariate map of harvested area and species richness. These maps were generated using the ‘raster’^30^ and ‘ggplot2’^29^ R packages with a ‘maps’ package^50^ world outline.

The four key crops in our study showed distinct global patterns in their production and environmental impacts (Figs. 2, 3, 4, 5). Wheat was primarily produced in temperate regions such as Europe, Central Asia, North America and parts of China, while sugar production was more geographically widespread, encompassing both temperate (sugar beet) and subtropical and tropical regions (sugarcane). Cocoa and oil palm were produced mainly in tropical countries, particularly in West Africa and Southeast Asia.

High levels of species richness and the harvested area of wheat predominantly overlapped in South-East Europe, Southern Russia and India (Fig. 2c). The environmental impact of wheat was primarily driven by the nitrogen fertiliser runoff (Nit) in Northern Europe and China (Fig. 2a). Greenhouse gas (GHG) emissions were the most prominent environmental impact factor in areas of Asia and the United States, while land-use pressures (LND) were the most significant issue in India.

For sugar (sugarcane and sugar beet combined), the overall distribution of impacts was similar to wheat, with high-impact areas mainly in Europe, plus in the Caribbean and Southeast Asia–Pacific (Fig. 3a, b). Here, land use (LND) was the main contributing impact (Fig. 3a). However, the overlap between species richness and sugar harvested area was spatially concentrated and especially strong in India and South America (Fig. 3c).

The environmental impacts of oil palm production were concentrated in tropical regions, especially in Southeast Asia & the Pacific Islands, West Africa and Central America (Fig. 4a). Therefore, the areas with the greatest species richness and harvested area have the highest environmental impacts (Fig. 4a, c). Greenhouse gas (GHG) impacts were mainly concentrated in Malaysia, while fertiliser runoff impacts (Nit) extended across Asia (Fig. 4a). High species-richness areas overlapped with regions of extensive oil palm harvesting in the tropics (Fig. 4c). However, GHG emissions were lower in Côte d’Ivoire than in Indonesia, while nitrogen (Nit) and phosphorus (Pho) fertiliser runoffs remained relatively constant in both areas (Fig. 4a). The region most impacted by oil palm was the area around Malaysia and Indonesia, with small areas in the Americas and West Africa also having high impact values (Fig. 4b). However, the land-use (LND) impacts of oil palm were more widespread than other impact factors (Fig. 4a).

Cocoa shared similar spatial patterns to oil palm but extended across a broader geographical range, with the highest impacts in Central America and the north of South America, Madagascar, West Africa, Southeast Asia and the Pacific islands (Fig. 5a, b). These areas experienced significant environmental pressures, though only GHG and LND values were available for cocoa (Fig. 5a). Land use-related biodiversity loss (LND) was the highest impact factor in most areas where cocoa was grown, with only small areas that show a high impact of GHG emissions.

### Comparing the environmental impacts of key ingredients in countries supplying the majority of UK imports

The UK sourced the majority of its wheat, sugar, palm oil, and cocoa from seven countries (Table 1). Much was sourced from the UK itself, including large amounts of sugar beet and wheat (Table 1), for which over 7 million and 14 million tonnes are supplied from domestic production, respectively (FAOSTAT; http://www.fao.org/faostat/en/#data/TM). Cocoa and palm oil were primarily sourced from tropical countries known to have high biodiversity. For cocoa, these countries were Côte d’Ivoire, Ghana, Nigeria and Cameroon and palm oil was primarily sourced from Indonesia and Malaysia. For palm oil, the proportion of the total production of these crops in these countries that was then exported to the UK was very low (~ 2%) yet fulfilled nearly half of UK consumption (Table 1).

**Fig. 6:**
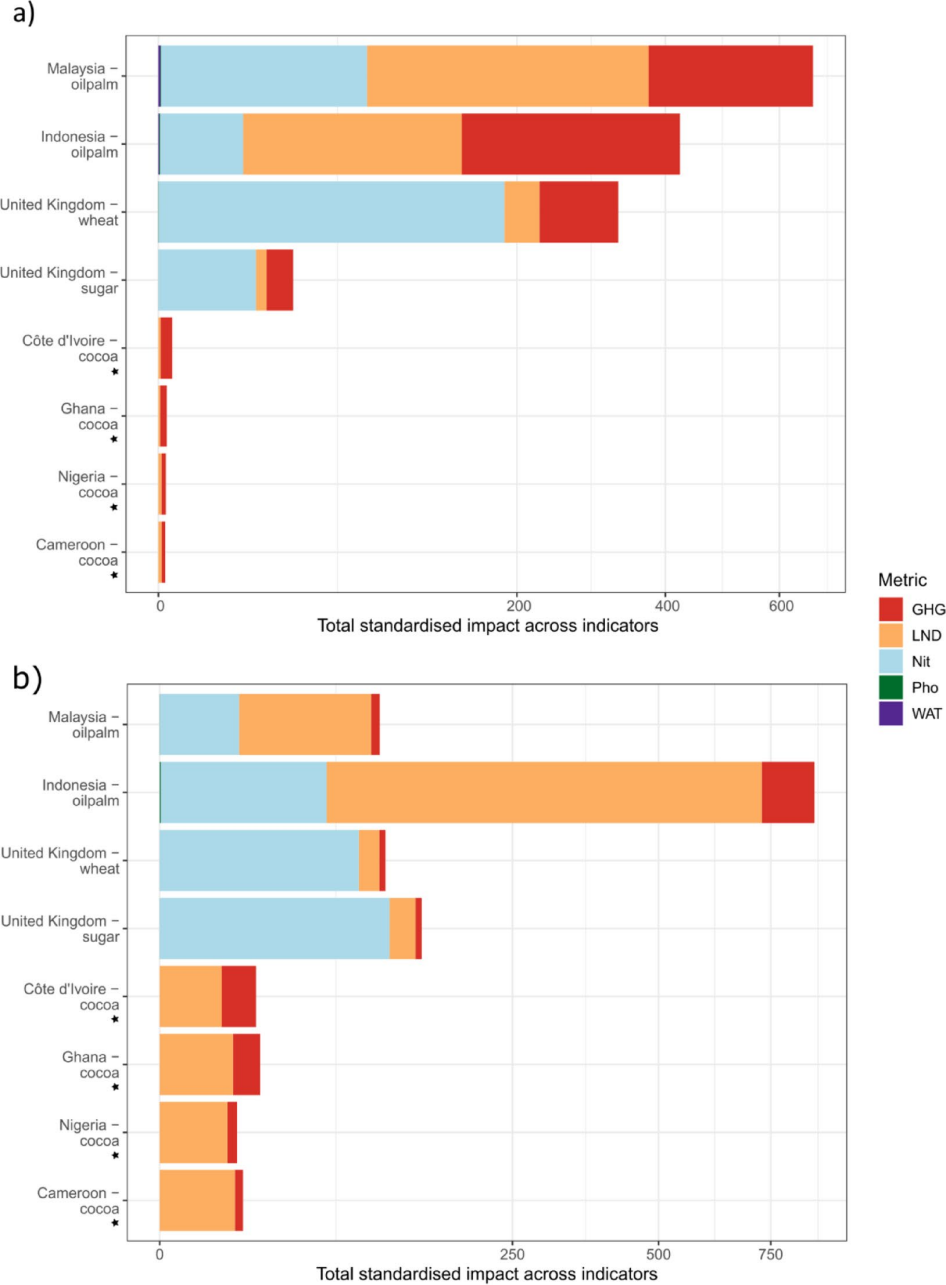
The environmental impact of growing the crops in countries that supplied at least 5% of the UK’s palm oil, sugar, wheat and cocoa. The bar charts show the standardised value of each environmental indicator generated for each crop, in total (**a**) and per tonne of crop (**b**). GHG, greenhouse gas emissions; LND, occupational land use biodiversity impacts; Nit, nitrogen fertiliser impacts on marine demersal fish and invertebrate species; Pho, phosphorus fertiliser impacts on freshwater fish species; and WAT, water debt. Note that, for cocoa, measures of impact were only available for GHG and LND; these are highlighted with stars. The x-axis has been square-root transformed to better visualise those countries with low impact scores.

Considering sugar, wheat and oil palm production, for which all five indicators could be calculated, the greatest impact was from oil palm production in Malaysia (especially for GHG and LND), followed by oil palm production in Indonesia, then wheat in the UK, for which Nit was the key impact metric (Fig. 6a). Sugar in the UK had the lowest total impact, most of which was attributable to Nit (Fig. 6a), though sugar had a greater per-tonne impact than wheat in the UK (Fig. 6b). For cocoa, only GHG and land use impact metrics were available. However, cocoa production showed much lower total impacts for these two metrics than the other three crops, with the highest total impact in Côte d’Ivoire (Fig. 6a).

More specifically, land-use impacts relating to Malaysian oil palm production were greatest, with a total standardised impact of 123, followed by Indonesian oil palm production at 74.5 (Fig. 6a). Per tonne, while other crops followed overall trends, Indonesian oil palm had a substantially greater per-tonne impact than Malaysian oil palm (Fig. 6b). Nit, LND, and GHG impacts were dominant in Indonesian oil palm impacts per tonne of crop (Fig. 6b).

The other crops showed a relatively low total land-use impact, with values between 0.005 and 1.9 (Ghanaian cocoa and UK wheat production respectively). GHGs were also a major contributor to crop environmental impacts; the greatest impacts again were from palm oil production, with Malaysian oil palm’s standardised total impact value at 74 and Indonesia’s at 42 (Fig. 6a). The GHG impact was a relatively strong contributor to UK wheat and sugar production impact too, with values of 9.7 for wheat and 1.1 for sugar (Fig. 6a). GHG impacts from cocoa production across the four producing countries included here were relatively low, with values ranging between 0.02 and 0.21 (Fig. 6a).

For the three crops for which impact information from nitrogen fertiliser was available (oil palm, wheat and sugar), the greatest impacts were from UK wheat production (186), followed by Malaysian oil palm (66) (Fig. 6a). Indonesian oil palm showed a much lower relative impact with a value of 11. For UK sugar production, nitrogen fertiliser impacts make up the greatest proportion of crop-related impact with a value of 14.7.

Both phosphorus and water-use impacts for oil palm, wheat and sugar were much lower than the other indicator metrics, with values for phosphorus ranging between 0.0000089 and 0.0014 and values for water between 0 (for UK sugar production) and 0.0056 (Fig. 6a).

Our findings suggest UK consumption of the focal crops has a potentially high contribution to environmental impacts overseas. Whilst not all of the evaluated crops are used in all processed treat foods such as biscuits, nor will all of the crop produced be used solely for the production of these kinds of food items, the UK is a large consumer of digestive biscuits and we can therefore refer to biscuits as an example product. A study investigating the variation of sugar and calorie content in cakes and biscuits sold in the UK in 2016 estimated that the average sugar content is 30/100g^51^. As a case-study example, this is 4.4 g per biscuit for dark chocolate digestive biscuits. Based on our estimate of UK biscuit consumption (see Introduction, calculated from public listing by McVities and data from statista.com and ONS.org.uk), this equates to over 52,000 tonnes of sugar, which is equivalent to 0.7% of total sugar produced in the UK. Similarly, for wheat, per biscuit, 96,000 tonnes of wheat is roughly equivalent to 0.7% of total wheat production. Quantities of palm oil in biscuits are generally undeclared. However, for digestive biscuits, roughly 5 g of chocolate per biscuit means approximately 60,000 tonnes of chocolate is consumed in biscuits alone per year. This is about a third of the total imported cocoa (assuming ratios of cocoa:chocolate are 1:1).

### Quantifying the extent to which threatened species could be impacted by crop production in countries that supply the UK

**Fig. 7:**
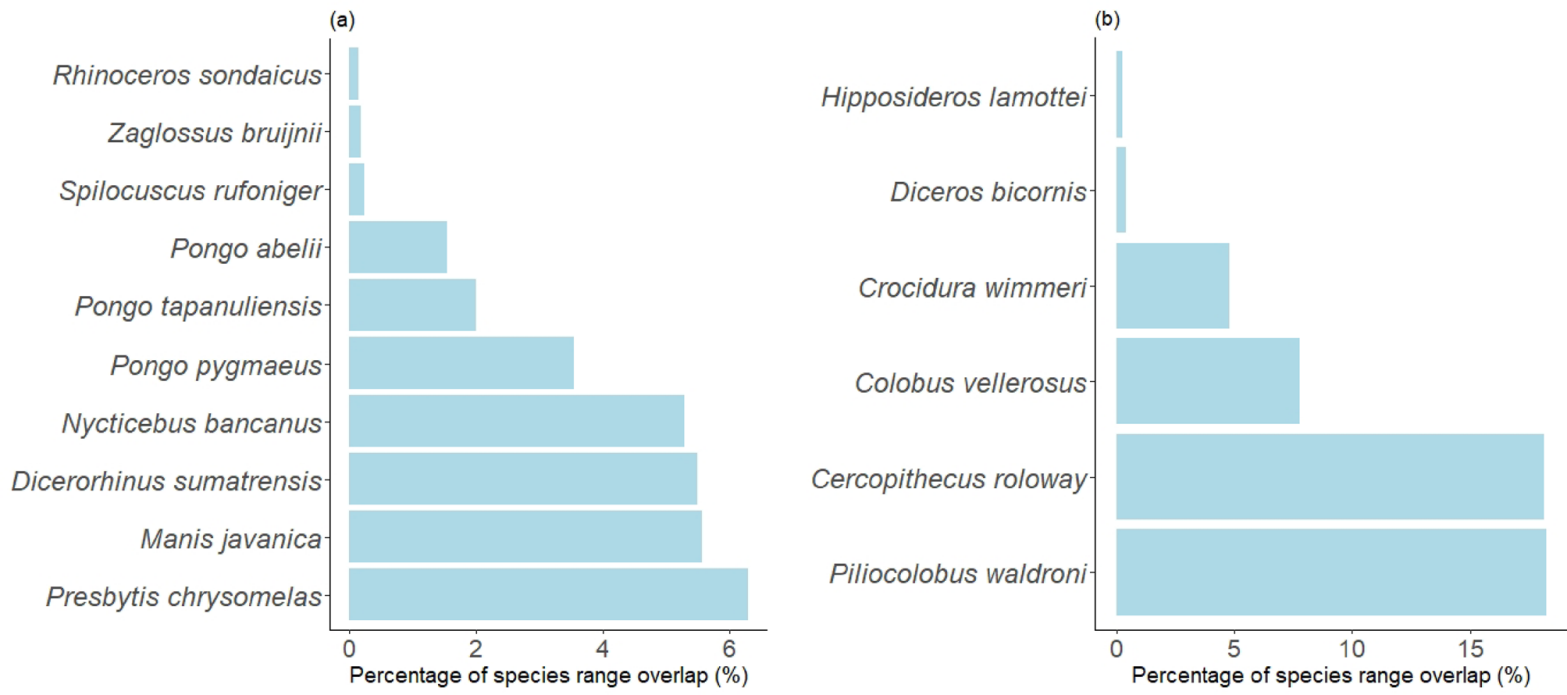
The percentage of Critically Endangered (CR) species’ ranges for (**a**) oil palm in Indonesia and (**b**) cocoa in Côte d’Ivoire which overlapped with areas producing each crop. Species common names are as follows, for reference: *Rhinoceros sondaicus:* Javan rhinoceros*, Zaglossus bruijnii:* Western long-beaked echidna, *Spilocuscus rufoniger:* Black-spotted cuscus, *Pongo abelii:* Sumatran orangutan, *Pongo tapanuliensis:* Tapanuli orangutan, *Pongo pygmaeus:* Bornean orangutan, *Nycticebus bancanus:* Bangka slow loris, *Dicerorhinus sumatrensis:* Sumatran rhinoceros, *Manis javanica:* Sunda pangolin, *Presbytis chrysomelas:* Sarawak surili*, Hipposideros lamottei:* Lamotte’s roundleaf bat, *Diceros bicornis:* black rhinoceros, *Crocidura wimmeri:* Wimmer’s shrew, *Colobus vellerosus:* ursine colobus, *Cercopithecus roloway:* Roloway monkey, *Piliocolobus waldroni:* Miss Waldron’s red colobus.

**Fig. 8:**
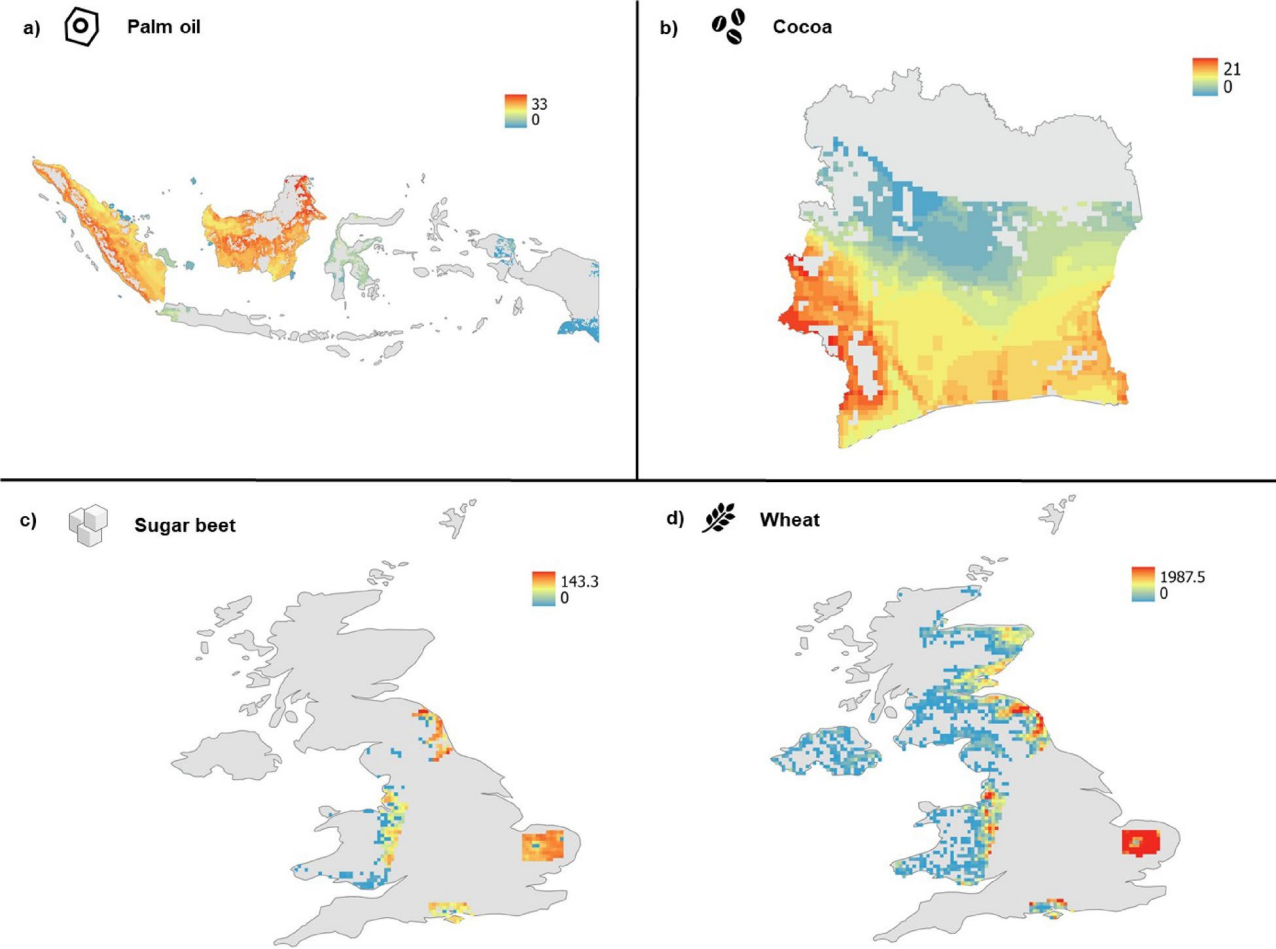
Maps of threatened species richness (Critically Endangered—CR, Endangered—EN, and Vulnerable—VU) with crop production areas for (**a**) oil palm in Indonesia and (**b**) cocoa in Côte d’Ivoire. For (**c**) and (**d**) the spatial overlap with crop production and the red squirrel’s (*Sciurus vulgaris*) range in the UK is shown for sugar beet and wheat. Values for (**a**) and (**b**) are the number of critically endangered species whereas for (**c**) and (**d**) they show the area of crop production in hectares. These maps were produced using Google Earth Engine^48^, ArcGIS Pro version 3.5^52^, and basemaps made with Natural Earth version 4.1.0.

There is considerable overlap of palm oil and cocoa production with threatened species’ ranges (species classified as Critically Endangered—CR, Endangered—EN, or Vulnerable—VU, by the IUCN). For Indonesian palm oil, the ranges of ten Critically Endangered species of mammal overlapped with Indonesian oil palm production (Fig. 7a). The largest recorded overlap for this highest category of threat (CR) was for *Presbytis chrysomelas* (6.3%)*,* the Bornean banded langur, closely followed by *Manis javanica*—the Sunda pangolin (Fig. 7a). The overlap with threatened species (CR, EN and VU) was greatest in western Indonesia, though the distribution of overlap across the region was patchy (Fig. 8a). For cocoa, the most notable overlap of species ranges with crop production was in southern Côte d’Ivoire (Fig. 8b). *Piliocolobus waldroni*—Miss Waldron’s red colobus—had the highest overlap with areas of cocoa production here (~ 18%) (Fig. 7b), closely followed by *Cercopithecus roloway*—the Roloway Monkey- and both species ranked much higher for overlap than the four other Critically Endangered (CR) species (Fig. 7b).

Several hotspots of wheat and sugar beet production overlapped with the known range of *Sciurus vulgaris* (red squirrel; Fig. 8), which is a species already pressured by habitat loss, as well as competition with invasive species and disease, and is classed as ‘near threatened’ in the UK (IUCN, 2021; Mathews and Harrower, 2020). The overlap between sugar, wheat, and the red squirrel varied by crop, with hotspots for sugar (Fig. 8c) and a more widespread distribution for wheat (though East Anglia was a hotspot region for this crop; Fig. 8d).

## Discussion

Previous studies have shown that the impact of crop production can vary depending on where and how it is produced^14,53^. However, these impacts are rarely spatially explicit and infrequently contain estimates of how biodiversity will be impacted, often focusing more on indicators such as greenhouse-gas emissions, land-use change or water use^54–56^. Not only does the impact matter for biodiversity conservation, but biodiversity is also critical for the health of ecosystems and, hence, for a sustainable and resilient agricultural system which heavily relies on ecosystem service provision^57^. Here, we quantified and mapped the biodiversity impacts associated with the production of palm oil, cocoa, sugar, and wheat. We focus on the four crops in our study: (i) given their dominance in ingredient lists for many luxury and ‘treat’ foods; (ii) to demonstrate how to make a first estimation of global biodiversity impacts for widespread and common crops; and (iii) to emphasise the need for biodiversity and broader, complementary environmental impact data to be included in sustainability assessments. We identified the global-scale biodiversity impacts associated with the crops grown to produce key ingredients in many luxury and ‘treat’ foods. We show that the demand for food, including these crops, has strong and wide-reaching environmental impacts that vary spatially and according to country of origin. Importantly, we show the potential biodiversity impacts that are not often evaluated in environmental impact studies. By assessing the spatial variation in several biodiversity-based impact indicators (i.e. not focusing solely on forest loss^58^), we also explored impacts on threatened species as a potential mode of communicating biodiversity impacts to consumers. However, this is currently limited by lack of knowledge of where specific crops for particular products are sourced from within a country.

In line with previous work^59,60^, we identify strong variability in impacts according to crop, region, and type of indicator. This highlights the importance of assessing multiple metrics for evaluating the biodiversity impacts of different foods. For instance, we show a distinction between hotspots of environmental impact and areas of strong overlap between harvested area and high species richness (Figs. 2, 3, 4, 5). This emphasises a need for complementary information, such as the amounts of a given crop being sourced from an area via international trade (Table 1), and its overlap with threatened species’ ranges (Fig. 7) to investigate how impacts vary spatially within and among regions. At this time, the coarse resolution of the impact maps we were able to produce at the global scale makes it challenging to identify specific areas at the local scale where impacts may be particularly high or low or to provide precise local-scale estimates (see Fig. 1 for data summary information). This makes providing conservation recommendations challenging, highlighting the need for finer-resolution data.

There remains a lack of transparency in global supply chains, especially those used by businesses creating products like biscuits on an industrial scale. Herein lies a major issue with quantifying and communicating biodiversity impacts associated with commonly consumed food products. Without improved supply-chain transparency, and similarly clear food labelling, the case studies and methods used in our study provide a means to estimate biodiversity impact but have an unavoidable level of uncertainty. For example, we found that the UK sources some of the focal crops from relatively low-impact regions. The proportion of a partner country’s production of the focal crops supplied to the UK are low in most cases (except cocoa from Ghana, which represents 9% of the country’s production) (Table 1). This could lead us to believe that the UK’s environmental impact on other countries is low. However, the reality is more complex. For instance, whilst the UK’s cocoa imports represent only ~ 5% of Côte d’Ivoire’s overall cocoa production (Table 1), we do not know from *where* the UK is sourcing the cocoa *within* Côte d’Ivoire and from which type of farming system. This 5% could represent the cocoa with the greatest impact, wherein choosing a product without this ingredient could make a large difference, or could represent cocoa farmed sustainably with low biodiversity impact, wherein its inclusion in a food item might be less of a concern to the consumer. There are attempts to model these supply chains in finer detail (e.g., the TRASE^61^ initiative for tracing deforestation and commodity trade pathways), but the data are limited. Additionally, traceability often only extends to distribution warehouses rather than the smallholder farmers that supply them. Thus, the 5% sourced from Côte d’Ivoire could mean UK demand is responsible for a relatively small proportion of Côte d’Ivoire’s potential biodiversity impacts associated with cocoa or a much larger proportion, hidden behind a lack of farm-specific or smaller spatial scale origin-tracing data^62^. This particular issue emphasises the importance of spatialising measures of environmental impact, to better capture the origin and endpoint of impacts. Either way, biscuits and other sweet treats within which these crops are staples could provide an opportunity for individuals to make alternative choices to reduce demand for impactful crops. For instance, consumers could reduce their consumption of sweet treats or choose options with a potential for lower impact (e.g., an option without chocolate, a different oil, or reduced sugar). Nevertheless, there is not always a clear ‘optimal’ consumer choice, especially as food labels do not often list the origin of each of the individual ingredients in a product.

Although farm-specific crop origins are difficult to trace, we found that a number of Critically Endangered species are at risk from habitat loss via agricultural development in countries where the UK sources the focal crops (Fig. 7). Endangered species can be the ‘face’ of a product’s impact, as is the case for oil palm, commonly linked to the loss of orangutan habitat and mentioned specifically in information on some products free of palm oil. While Indonesian orangutans (*Pongo pygmaeus* and *Pongo abelii*) are often the ‘face’ of oil palm’s impact, ‘icons of wild nature’ according to Spehar et al.^63^, our findings reveal that lesser-known species like the *Presbytis chrysomelas* and the Sunda pangolin are facing significant threats, urging a broader awareness of biodiversity loss. *Presbytis* primates are relatively understudied, but the *chrysomelas* species we identify as having strong overlap with oil palm is endemic to Borneo and is losing its forest habitat to logging and conversion to agriculture. This is of particular concern because these monkeys cannot survive in deforested habitats and are unlikely to cross between forest patches^57^. Whilst our study cannot be used to accurately pinpoint overlap between threatened species and specific crops, we can infer from this that any overlap has the potential for direct impact via habitat loss, but also indirect effects on humans. Recent studies further support this, highlighting that even palm oil production labelled as ‘sustainable’ has been associated with widespread habitat loss for orangutans and other species (e.g. see Gatti and Velichevskaya^64^).

For cocoa, we identified two primate species for which almost 20% of their range overlapped with crop production—*Cercopithecus roloway* and *Piliocolobus waldroni.* Whilst certain species can adapt to changing landscapes and coexist alongside agricultural production (e.g., generalist species and species able to survive in smaller patches^63,65^), many cannot, and around 90% of Critically Endangered mammals have agricultural land listed as a major driver of their extinction risk^66^. The red colobus monkey, which had the greatest overlap with cocoa production in Côte d’Ivoire of all Critically Endangered species analysed (Fig. 7), has been noted as potentially extinct in previous years and is endemic only to the two major cocoa producers - Côte d’Ivoire and Ghana^67^. These case studies reveal the ‘hidden’ impacts of global trade in producer countries, often unknown to consumers purchasing the products and difficult for sellers and researchers to quantify due to lengthy and opaque supply chains^68^.

Our understanding of the relative sustainability of different food products is currently limited by the differing baselines of biodiversity records. Although the UK has no threatened mammal species according to the IUCN Red List, this does not mean that there are no threats to UK biodiversity. Due to the country’s history of industrialisation and agricultural intensification, it is likely that threatened mammalian biodiversity was lost before Red List data were collected. This bias is present in many countries with long histories of intense land-use change and influences our understanding of environmental impacts, which appear stronger in the tropics where biodiversity is higher (e.g., as discussed in Ellis et al.^69^). This highlights the potential for injustice in making sustainable land-use decisions: do we source ingredients from places that have already benefited economically through habitat loss from previous agricultural development, rather than potentially less economically developed regions with higher biodiversity?

Our study is limited by the necessary assumption that a species’ range overlapping with an area of high crop production is placing that species at risk. This is based on the fact that many species on the IUCN Red List are listed as threatened due to cropland expansion and habitat loss for agriculture, and that previous research has shown lower biodiversity in cropland than natural habitats^70,71^. Importantly, there are large differences in the production systems associated with the crops examined in this study. For instance, cocoa can be grown on small plots alongside other vegetation (shaded cocoa) which can enhance tree cover and ecosystem services^72^(though note this depends on shade tree type, land-use history, and other characteristics—e.g., Blaser-Hart et al.^73^ and Maney et al.^74^), whilst oil palm tends to be grown in vast areas of monoculture. These distinctions are lost in global-scale analyses such as ours. Additionally, only data for LND and GHG impacts were available for cocoa. As a result, we are underestimating the total environmental impacts of this crop, especially as fertilisers are recommended by Ghana’s cocoa board and associated with yield improvements, for example^75^. According to water footprints of cocoa grown in Colombia, some cocoa varieties are more water-efficient than others, providing greater yields with the same quantities of rainfall^76^, highlighting a future research need for variety-specific biodiversity-impact measurement, as well as crop-specific studies. Whilst beyond the scope of our study, we anticipate that several of the metrics we used could be developed to incorporate more facets of biodiversity (e.g., functional and phylogenetic diversity).

Our analysis of products consumed in the UK highlights complexities inherent in evaluating sustainability of snacks like digestive biscuits. Sustainably sourced food is increasingly high on the agenda of consumers in countries including the UK, where there is an increasing trend towards plant-based diets^77^. However, making diets more sustainable comes with several challenges, such as the cost, political economy, cultural norms, justice, and governance issues highlighted by Béné and Abdulai^78^. Furthermore, the complexity inherent in ensuring the overall environmental sustainability of products, particularly when considering biodiversity, makes it difficult to simply and effectively communicate the environmental impacts of food production to consumers. In the future, when more field data on species’ responses to specific crops are available and supply chains are more transparent, consumers could be informed of the specific impacts of each dietary decision more easily. For now, by mapping crop-specific environmental impact hotspots and highlighting vulnerable species at risk according to their range overlap with areas of high levels of food crop production, we anticipate that this, and future studies expanding the scope to other commonly consumed food items, will help to reduce the disconnect between consumers and their biodiversity impacts beyond packaging. To date, there has been a consumer focus on reducing plastic waste to protect biodiversity. We propose that further education and industry initiatives are required to build on this established momentum towards more sustainable practices and to expand the focus to ingredient origins and how to make more sustainable food choices.

## Supplementary Information

Below is the link to the electronic supplementary material.

Supplementary Material 1

## Data Availability

The data used as inputs for the analyses in this manuscript were openly available for download, as described and cited in the manuscript. The code used for analyses are available from: https://github.com/CharlieOuthwaite/Biscuit_project.
