# Supplementary material for: Key crops for processed foods have spatially variable biodiversity impacts not captured in other environmental impact indicators
Source: Sci Rep. 2026 Jan 29;16:5450. doi: 10.1038/s41598-025-34850-2 (PMC12887044; doi:10.1038/s41598-025-34850-2)
Supplement: Supplementary file 1 — Supplementary Material 1 [file 41598_2025_34850_MOESM1_ESM.docx]

SUPPLEMENTARY INFORMATION

**Supporting Information S1:** Global-scale maps of environmental impact for each impact and crop type

We developed a custom function to create the bivariate map for total species richness and the four crop harvest maps. This function can be used to compare two environmental indicators, such as species richness and crop harvest distribution or greenhouse gas emissions, and to visualise the spatial relationships between these indicators. Whilst, ultimately, the function was not used to compare any of the five impact factors with species richness for our study (since some of the impact factors are based on the impact the crop has on the species in the cells), it is provided in code available on GitHub (see Data Availability Statement in manuscript) for reference, as we anticipate bivariate mapping will prove useful for communicating trade-offs to consumers, for instance. The function handled the resampling and projection alignment.

**Supporting Information S2:** The full list of source countries for the four ingredient crops investigated, with respect to UK imports/production. The UK has 169 unique trade partners for cocoa, 5 for palm oil, 9 for wheat, and 3 for sugarcane over the time period considered (2003-2013).

| **Crop** | **Partner** |
| --- | --- |
| cocoa | Côte d'Ivoire |
| cocoa | Ghana |
| cocoa | Nigeria |
| cocoa | Cameroon |
| cocoa | Indonesia |
| cocoa | Togo |
| cocoa | Papua New Guinea |
| cocoa | Ecuador |
| cocoa | Brazil |
| cocoa | Malaysia |
| cocoa | Dominican Republic |
| cocoa | Peru |
| cocoa | Venezuela (Bolivarian Republic of) |
| cocoa | Guinea |
| cocoa | Madagascar |
| cocoa | Sao Tome and Principe |
| cocoa | Sierra Leone |
| cocoa | Philippines |
| cocoa | Colombia |
| cocoa | United Republic of Tanzania |
| cocoa | Liberia |
| cocoa | Uganda |
| cocoa | Solomon Islands |
| cocoa | Haiti |
| cocoa | Equatorial Guinea |
| cocoa | Guatemala |
| cocoa | Vanuatu |
| cocoa | Trinidad and Tobago |
| cocoa | Democratic Republic of the Congo |
| cocoa | Jamaica |
| cocoa | Thailand |
| cocoa | Congo |
| cocoa | Mexico |
| cocoa | Bolivia (Plurinational State of) |
| cocoa | Honduras |
| cocoa | El Salvador |
| cocoa | Cuba |
| cocoa | Nicaragua |
| cocoa | Dominica |
| cocoa | Panama |
| cocoa | India |
| cocoa | Costa Rica |
| cocoa | Grenada |
| cocoa | United Kingdom |
| cocoa | Sri Lanka |
| cocoa | Central African Republic |
| cocoa | Ireland |
| cocoa | Gabon |
| cocoa | Belize |
| cocoa | Denmark |
| cocoa | France |
| cocoa | Netherlands |
| cocoa | Switzerland |
| cocoa | Luxembourg |
| cocoa | Belgium |
| cocoa | Guyana |
| cocoa | Germany |
| cocoa | Sweden |
| cocoa | Ethiopia |
| cocoa | Niger |
| cocoa | Turkey |
| cocoa | Bahrain |
| cocoa | Italy |
| cocoa | Samoa |
| cocoa | Algeria |
| cocoa | Suriname |
| cocoa | Fiji |
| cocoa | Benin |
| cocoa | Poland |
| cocoa | Spain |
| cocoa | Malta |
| cocoa | Iraq |
| cocoa | Austria |
| cocoa | Finland |
| cocoa | Kenya |
| cocoa | Singapore |
| cocoa | China, mainland |
| cocoa | Brunei Darussalam |
| cocoa | Kiribati |
| cocoa | South Africa |
| cocoa | Hungary |
| cocoa | China, Hong Kong SAR |
| cocoa | Slovenia |
| cocoa | Slovakia |
| cocoa | Pakistan |
| cocoa | Norway |
| cocoa | Senegal |
| cocoa | Czechia |
| cocoa | Argentina |
| cocoa | Myanmar |
| cocoa | Chile |
| cocoa | Serbia |
| cocoa | New Zealand |
| cocoa | Saint Lucia |
| cocoa | Nepal |
| cocoa | Tajikistan |
| cocoa | Israel |
| cocoa | Egypt |
| cocoa | Eswatini |
| cocoa | Portugal |
| cocoa | Yemen |
| cocoa | Uruguay |
| cocoa | Australia |
| cocoa | United Arab Emirates |
| cocoa | Canada |
| cocoa | Greece |
| cocoa | Cyprus |
| cocoa | United States of America |
| cocoa | Latvia |
| cocoa | Zimbabwe |
| cocoa | Lithuania |
| cocoa | Japan |
| cocoa | Republic of Korea |
| cocoa | Lebanon |
| cocoa | Armenia |
| cocoa | Lesotho |
| cocoa | Syrian Arab Republic |
| cocoa | Croatia |
| cocoa | China, Taiwan Province of |
| cocoa | Bulgaria |
| cocoa | North Macedonia |
| cocoa | Faroe Islands |
| cocoa | Maldives |
| cocoa | Saudi Arabia |
| cocoa | Sudan |
| cocoa | Georgia |
| cocoa | Somalia |
| cocoa | Palestine |
| cocoa | Afghanistan |
| cocoa | Antigua and Barbuda |
| cocoa | Mali |
| cocoa | Oman |
| cocoa | Bosnia and Herzegovina |
| cocoa | China, Macao SAR |
| cocoa | Democratic People's Republic of Korea |
| cocoa | Morocco |
| cocoa | Tunisia |
| cocoa | Iran (Islamic Republic of) |
| cocoa | Bangladesh |
| cocoa | Cambodia |
| cocoa | Mauritius |
| cocoa | Romania |
| cocoa | Ukraine |
| cocoa | Libya |
| cocoa | Barbados |
| cocoa | Seychelles |
| cocoa | Kazakhstan |
| cocoa | Russian Federation |
| cocoa | Albania |
| cocoa | Tonga |
| cocoa | Republic of Moldova |
| cocoa | Mauritania |
| cocoa | Kuwait |
| cocoa | Jordan |
| cocoa | Estonia |
| cocoa | Belarus |
| cocoa | Namibia |
| cocoa | Saint Vincent and the Grenadines |
| cocoa | Zambia |
| cocoa | Montenegro |
| cocoa | Burkina Faso |
| cocoa | Malawi |
| cocoa | Mongolia |
| cocoa | Kyrgyzstan |
| cocoa | Qatar |
| cocoa | Paraguay |
| cocoa | Azerbaijan |
| cocoa | Iceland |
| cocoa | Botswana |
| oilpalm | Angola |
| oilpalm | Gambia |
| oilpalm | Guinea-Bissau |
| oilpalm | Burundi |
| oilpalm | Rwanda |
| wheat | Bhutan |
| wheat | Uzbekistan |
| wheat | Eritrea |
| wheat | Turkmenistan |
| wheat | Chad |
| wheat | Saint Kitts and Nevis |
| wheat | Micronesia (Federated States of) |
| wheat | Bahamas |
| wheat | Cabo Verde |
| sugarcane | Mozambique |
| sugarcane | Lao People's Democratic Republic |
| sugarcane | Viet Nam |

**Supporting Information S3:** The proportion of species ranges found within the producing areas of each focal crop for cocoa and palm oil, for all threatened categories; critically endangered, endangered and vulnerable.

**Species Proportion of range overlapping with cocoa**

CR 1.737745806

Cercopithecus roloway 18.14465678

Colobus vellerosus 7.776456436

Crocidura wimmeri 4.786376194

Diceros bicornis 0.424547275

Hipposideros lamottei 0.280666585

Piliocolobus waldroni 18.22056242

EN 1.602304798

Cephalophus jentinki 1.848831238

Cercocebus lunulatus 16.56951844

Cercopithecus diana 2.285007095

Colobus polykomos 1.584091804

Hylomyscus baeri 15.60243325

Pan troglodytes 1.823535297

Phataginus tricuspis 1.04708128

Piliocolobus badius 5.239959636

Rhinolophus guineensis 0.322823478

Smutsia gigantea 1.346598671

VU 1.377189198

Acinonyx jubatus 0.000324

Caracal aurata 1.106477644

Cephalophus zebra 1.870382911

Cercocebus atys 1.484133462

Cercopithecus lowei 13.11834222

Genetta bourloni 0.632105677

Hipposideros marisae 2.055667593

Liberiictis kuhni 2.617050657

Loxodonta africana 0.334180015

Panthera leo 0.003009215

Panthera pardus 0.301628366

Phataginus tetradactyla 1.707986238

Poiana leightoni 1.679827709

Procolobus verus 9.171462163

**Species Proportion of range overlapping with palm oil**

CR 4.60725202

Ailurops melanotis 0

Axis kuhlii 0

Dendrolagus mayri 0

Dendrolagus pulcherrimus 0

Dicerorhinus sumatrensis 5.488618939

Macaca nigra 0

Macaca pagensis 0

Manis javanica 5.560714376

Nycticebus bancanus 5.303764116

Pongo abelii 1.544447036

Pongo pygmaeus 3.544331302

Pongo tapanuliensis 1.991302617

Presbytis chrysomelas 6.296612495

Presbytis potenziani 0

Pteropus aruensis 0

Rhinoceros sondaicus 0.142583446

Simias concolor 0

Spilocuscus rufoniger 0.236249499

Spilocuscus wilsoni 0

Tarsius tumpara 0

Uromys boeadii 0

Zaglossus attenboroughi 0

Zaglossus bruijnii 0.182994142

EN 4.072207433

Acerodon humilis 0

Bos javanicus 6.712931063

Bubalus depressicornis 0.858825805

Bubalus quarlesi 0.694777623

Bunomys coelestis 0

Bunomys prolatus 0

Catopuma badia 0.673688313

Cuon alpinus 0.576590875

Cynogale bennettii 8.544786028

Dendrolagus mbaiso 0

Echiothrix leucura 0

Elephas maximus 2.605883053

Hipposideros coxi 15.2976207

Hylobates abbotti 6.192726689

Hylobates agilis 10.21398179

Hylobates albibarbis 2.771994912

Hylobates funereus 5.373778743

Hylobates klossii 0

Hylobates lar 6.045689195

Hylobates moloch 0.238672488

Hylobates muelleri 2.906089745

Hylopetes sipora 0

Iomys sipora 0

Kadarsanomys sodyi 0.21925724

Macaca maura 0

Macaca nemestrina 6.225496347

Macaca siberu 0

Mallomys gunung 0

Margaretamys christinae 0

Maxomys wattsi 0

Melomys aerosus 0

Melomys bannisteri 0

Melomys caurinus 0

Melomys fraterculus 0

Melomys talaudium 0

Nasalis larvatus 4.448717882

Neopteryx frosti 0

Nesoromys ceramicus 0

Nycticebus coucang 9.124960002

Nycticebus hilleri 4.951929703

Nyctimene rabori 0

Panthera tigris 3.043396829

Paraleptomys rufilatus 0

Paulamys naso 0

Phalanger alexandrae 0

Presbytis canicrus 1.728293699

Presbytis comata 0.296251024

Presbytis melalophos 7.621058669

Presbytis sabana 16.14224928

Presbytis siberu 0

Presbytis sumatranus 9.93469092

Prosciurillus weberi 3.299855533

Pteromyscus pulverulentus 6.241394779

Pteropus melanopogon 0

Rattus hainaldi 0

Rattus simalurensis 0.000298055

Rhinolophus belligerator 0.026068932

Rhinolophus proconsulis 19.56088418

Rhynchomeles prattorum 0

Suncus mertensi 0

Sus verrucosus 0.294911276

Symphalangus syndactylus 7.626683582

Tarsius pelengensis 0

Tarsius sangirensis 0

VU 4.855865379

Acerodon celebensis 0.966805594

Acerodon mackloti 0

Ailurops ursinus 0.955095881

Arctictis binturong 5.962558174

Arielulus cuprosus 9.097165695

Boneia bidens 1.106995953

Bunomys fratrorum 0

Callosciurus melanogaster 0

Capricornis sumatraensis 3.171418675

Chaerephon johorensis 16.9310097

Chiropodomys karlkoopmani 0

Coelops robinsoni 1.144857483

Dendrolagus inustus 0.299231402

Dendrolagus stellarum 0

Dendrolagus ursinus 0.204629109

Diplogale hosei 0.402704695

Dobsonia emersa 0

Dorcopsis luctuosa 0.240609441

Dyacopterus brooksi 9.313060977

Echiothrix centrosa 0.355072164

Emballonura serii 0

Eropeplus canus 0.019894182

Haeromys minahassae 0.231534543

Haeromys pusillus 3.716671161

Harpyionycteris celebensis 1.013064053

Helarctos malayanus 6.102568063

Hipposideros orbiculus 22.11948661

Hipposideros ridleyi 13.58870102

Hipposideros sorenseni 0

Hylomys parvus 0.109859011

Hyosciurus ileile 0.176732753

Kerivoula flora 0

Komodomys rintjanus 0

Leopoldamys siporanus 0

Macaca fascicularis 5.29972584

Macaca hecki 0.32259682

Macaca nigrescens 0

Macaca ochreata 1.078333613

Macaca tonkeana 1.511731304

Macrogalidia musschenbroekii0.710331736

Maxomys inflatus 2.112981286

Maxomys pagensis 0

Maxomys rajah 6.220687432

Maxomys whiteheadi 6.237186241

Megaerops kusnotoi 0.163097259

Megaerops wetmorei 2.123261948

Microperoryctes aplini 0.116938587

Microperoryctes murina 0

Murina aenea 6.866525398

Murina rozendaali 4.503947773

Neofelis diardi 1.97707272

Nycteris javanica 0.132045308

Nycticebus borneanus 2.554613059

Nycticebus kayan 5.486901135

Nycticebus menagensis 4.462359123

Nyctimene keasti 0

Nyctimene minutus 0

Panthera pardus 0.14156953

Petinomys genibarbis 8.308153736

Petinomys lugens 0

Petinomys setosus 9.397197307

Petinomys vordermanni 7.133669435

Phalanger matabiru 0

Pithecheir melanurus 0

Presbytis femoralis 19.52886442

Presbytis frontata 1.909671704

Presbytis hosei 2.604462961

Presbytis mitrata 7.383033475

Presbytis natunae 3.450395699

Presbytis rubicunda 4.036312281

Presbytis thomasi 4.008426361

Pseudochirops coronatus 0.109748606

Pseudochirulus schlegeli 0.138268339

Pteropus caniceps 0

Pteropus chrysoproctus 0

Pteropus melanotus 0.217319209

Pteropus ocularis 0

Pteropus pohlei 0

Pteropus temminckii 0

Rattus hoogerwerfi 0

Rattus lugens 0

Rattus richardsoni 0

Rheithrosciurus macrotis 4.434232424

Rhinolophus canuti 0

Rhinolophus madurensis 0

Rousettus spinalatus 10.20638492

Rubrisciurus rubriventer 1.010178296

Rusa timorensis 0.015815154

Rusa unicolor 6.171455755

Spilocuscus papuensis 0

Sundamys maxi 0.05956195

Sundasciurus fraterculus 0

Sus barbatus 4.438437381

Syconycteris carolinae 0

Taeromys taerae 0

Tarsius bancanus 4.598914304

Tarsius dentatus 1.010905616

Tarsius tarsier 0.964060119

Thylogale browni 0.169068189

Thylogale brunii 0.037785974

Trachypithecus auratus 0.131538664

Trachypithecus cristatus 6.214560501

Tupaia chrysogaster 0

Zaglossus bartoni 0.047232353
